# An ALE Meta-Analysis of Specific Functional MRI Studies on Subcortical Vascular Cognitive Impairment

**DOI:** 10.3389/fneur.2021.649233

**Published:** 2021-09-22

**Authors:** Wenwen Xu, Yu Song, Shanshan Chen, Chen Xue, Guanjie Hu, Wenzhang Qi, Wenying Ma, Xingjian Lin, Jiu Chen

**Affiliations:** ^1^Department of Neurology, The Affiliated Brain Hospital of Nanjing Medical University, Nanjing, China; ^2^Department of Radiology, The Affiliated Brain Hospital of Nanjing Medical University, Nanjing, China; ^3^Institute of Brain Functional Imaging, Nanjing Medical University, Nanjing, China; ^4^Institute of Neuropsychiatry, The Affiliated Brain Hospital of Nanjing Medical University, Nanjing, China

**Keywords:** subcortical vascular cognitive impairment, resting state, ALE, amplitude of low-frequency fluctuation, regional homogeneity, functional connectivity

## Abstract

**Background:** Subcortical vascular cognitive impairment (sVCI), caused by cerebral small vessel disease, accounts for the majority of vascular cognitive impairment, and is characterized by an insidious onset and impaired memory and executive function. If not recognized early, it inevitably develops into vascular dementia. Several quantitative studies have reported the consistent results of brain regions in sVCI patients that can be used to predict dementia conversion. The purpose of the study was to explore the exact abnormalities within the brain in sVCI patients by combining the coordinates reported in previous studies.

**Methods:** The PubMed, Embase, and Web of Science databases were thoroughly searched to obtain neuroimaging articles on the amplitude of low-frequency fluctuation, regional homogeneity, and functional connectivity in sVCI patients. According to the activation likelihood estimation (ALE) algorithm, a meta-analysis based on coordinate and functional connectivity modeling was conducted.

**Results:** The quantitative meta-analysis included 20 functional imaging studies on sVCI patients. Alterations in specific brain regions were mainly concentrated in the frontal lobes including the middle frontal gyrus, superior frontal gyrus, medial frontal gyrus, and precentral gyrus; parietal lobes including the precuneus, angular gyrus, postcentral gyrus, and inferior parietal lobule; occipital lobes including the lingual gyrus and cuneus; temporal lobes including the fusiform gyrus and middle temporal gyrus; and the limbic system including the cingulate gyrus. These specific brain regions belonged to important networks known as the default mode network, the executive control network, and the visual network.

**Conclusion:** The present study determined specific abnormal brain regions in sVCI patients, and these brain regions with specific changes were found to belong to important brain functional networks. The findings objectively present the exact abnormalities within the brain, which help further understand the pathogenesis of sVCI and identify them as potential imaging biomarkers. The results may also provide a basis for new approaches to treatment.

## Introduction

Cerebral small vessel disease (CSVD), referring to the series of neuropathological processes related to heredity and age, continuously damages the small perforating arteries, arterioles, capillaries, and venules (1). The cerebral white and deep gray matter are inevitably damaged, which further results in the onset of occult cognitive impairment (1). Vascular cognitive impairment (VCI), including VCI non-dementia (VCIND), vascular dementia (VD), and mixed dementia with VCI, is a potentially preventable and treatable cognitive impairment (2). Accounting for about 36–67% of VCI, subcortical vascular cognitive impairment (sVCI) caused by CSVD needs early recognition and intervention, which is the key to reducing the incidence of VD (3). Topics surrounding sVCI such as cognitive impairment, imaging features, and biological markers have gradually become the focus of today's international stroke and domestic research (4). It is well known that dementia is a group of syndromes involving memory loss, judgment, reasoning, mood changes, behavior, and communication (5). However, compared with AD, the cognitive characteristics of sVCI, involve the domains of executive function impairment and attention deficit, rather than more prominent memory impairment (6). It is reasonable to deduce that the main damaged brain area in AD and the main damaged brain area in sVCI are dissimilar because of the difference in cognitive impairment between the two neurodegenerative diseases, which is a point worthy of further study.

CSVD can be divided into three disease subtypes as follows (7). Subtype 1 is composed of sporadic arteriolosclerosis accompanied by aging and several vascular risk factors, which include systemic arterial hypertension and diabetes mellitus (7). Subtype 2 is defined by cerebral amyloid angiopathy (CAA) linked to hereditary (7). Subtype 3 refers to all inherited or genetic CSVD subtypes without CAA (7). Among them, the most common disease is cerebral autosomal dominant arteriopathy with subcortical ischemic strokes and leukoencephalopathy (8). The common feature of the different subtypes is hemorrhage or ischemia of the subcortical small cerebral vessels. Therefore, it is necessary to clarify the characteristics and types of CSVD by searching original studies and conducting comprehensive quantitative analysis.

In terms of neuroimaging, CSVD appears as white matter lesions, lacunar infarcts, enlarged perivascular spaces, and cerebral microbleeds (8). Resting-state functional magnetic resonance imaging (fMRI) has made it possible for researchers to detect alterations in brain functional networks and local spontaneous neuronal activity, which provides new insight into the pathogenesis of neurological diseases (9). At present, there are three reliable technical means widely used in fMRI, including the amplitude of low-frequency fluctuation (ALFF), regional homogeneity (ReHo), and functional connectivity (FC) (10–12). The ALFF is used to describe spontaneous regional brain activity and ReHo is used to demonstrate the consistency of brain activity (10). FC reveals whether there is connectivity disruption or compensation in the brain regions (12). In the field of neuroimaging, the known networks for cognitive impairment include the default mode network (DMN), the executive control network (ECN), and the visual network (VN). Different networks interact and coordinate with each other to maintain the cognitive level (13). When cognitive impairment occurs, these networks are undoubtedly affected, and vice versa (13). Since the clinical cognitive impairment of sVCI involves multiple aspects, impairment in different networks is likely to occur.

Anatomical Likelihood Estimation (ALE) is a coordinate-based meta-analysis method whose principle is that a 3D Gaussian probability distribution is derived from each coordinate of the research (14). ALE maps are formed by synthesizing the distribution of all eligible studies with a threshold of *p* < 0.05 (15). ALE has demonstrated statistically significant results in studies of various neurological or psychiatric disorders (16). There are no original articles on sVCI in the study of resting-state FC and spontaneous neuronal activity. However, a rare meta-analysis was conducted on specific functional alterations of the brain regions in sVCI patients. Therefore, it is necessary to summarize the previously published articles on sVCI caused by CSVD to determine the specific cognitive domains and brain regions damaged using the ALE algorithm. This study was conducted to investigate the potential targets of sVCI and further discover the pathogenesis. Hence, we made assumptions that in sVCI: (1) specific brain functional markers associated with cognitive impairment would be revealed in sVCI patients and (2) these specific markers would belong to important networks whose interruption has been associated with cognitive decline.

## Method

The meta-analysis of the present studies was based on the PRISMA statement guidelines.

### Literature Retrieval and Research Selection

#### Retrieval Strategies

**Figure 1:**
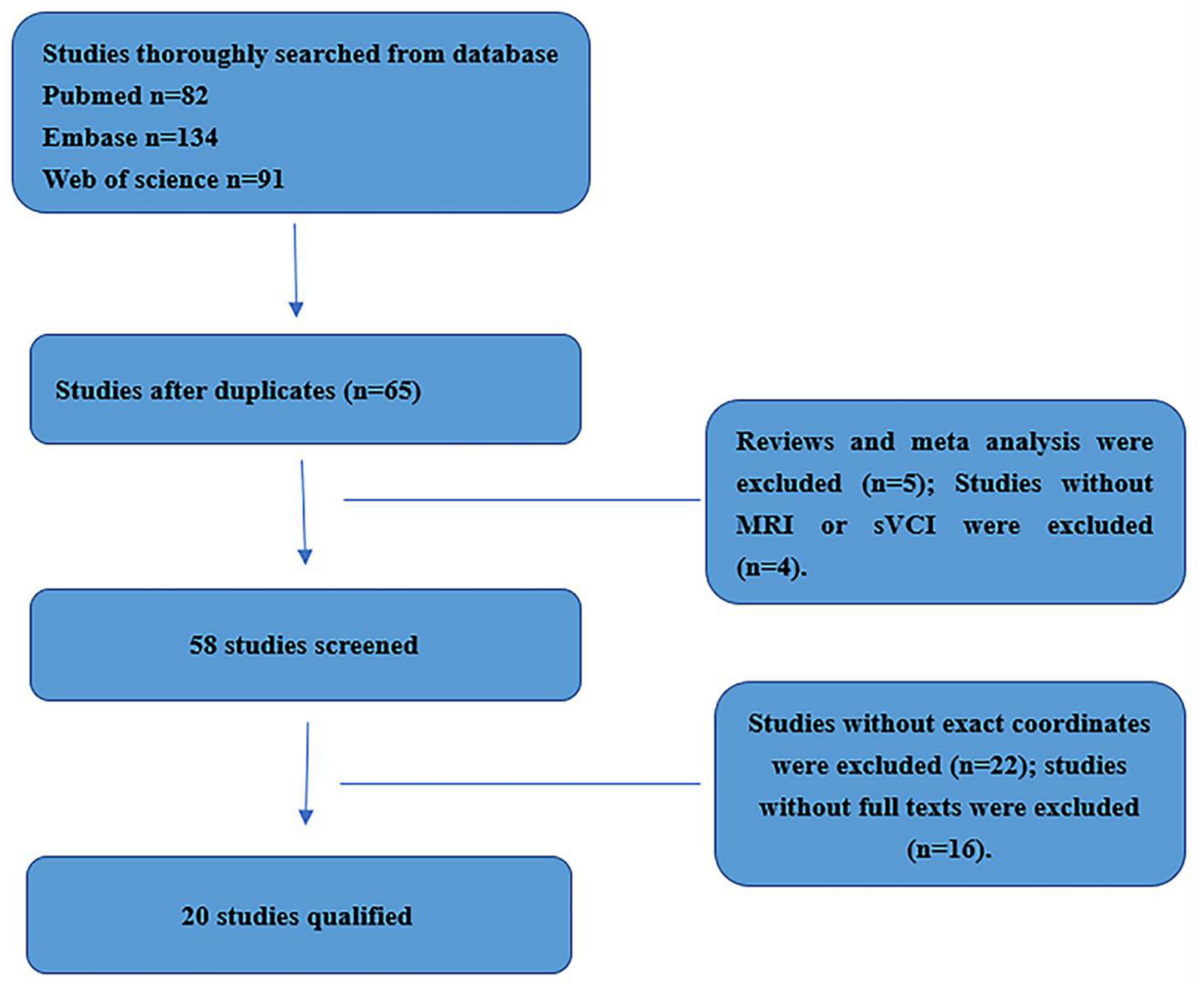
The flowchart of meta-analysis.

Two researchers thoroughly and systematically searched the PubMed, Embase, and Web of Science databases using the keywords (1) (“functional magnetic resonance imaging [MeSH] OR “RESTING STATE” [MeSH]) AND (“subcortical vascular cognitive impairment” [MeSH]) AND (“Functional connectivity”); (2) (“functional magnetic resonance imaging [MeSH] OR “RESTING STATE” [MeSH]) AND (“subcortical vascular cognitive impairment” [MeSH] AND “regional homogeneity”); (3) (“functional magnetic resonance imaging” [MeSH] OR “RESTING STATE” [MeSH]) AND (“subcortical vascular cognitive impairment” [MeSH] AND (“amplitude of low frequency fluctuation”) (Supplementary Table 1). Figure 1 shows the flowchart of the literature search and selection strategy.

#### Inclusion and Exclusion Criteria

##### Inclusion Criteria

The inclusion criteria were (1) lacunar infarction (LI), cerebral microinfarct, hemorrhage (CMB), leukoencephalopathy (WML), lacunae, and enlarged perivascular gaps on head magnetic resonance imaging (MRI) (17); (2) diagnostic criteria for CSVD based on CSVD expert consensus (18); and (3) the results of the assessment of all the enrolled patients were within the range of cognitive impairment.

##### Exclusion Criteria

The exclusion criteria were (1) existing or past infarction, hemorrhage, and derma in 1/3 of the cerebral lobe; (2) obvious cerebral artery stenosis or occlusion (18); (3) other diseases of the brain that cause leukinopathy (e.g., hypoglycemia, poisoning, and immune disorders (18)); (4) patients with intracranial tumors and major organ dysfunction (18); and (5) patients with dementia, mental symptoms, audiovisual impairment, and the inability to cooperate (18).

##### Article Requirements

The articles needed to contain resting-state functional MRI and present Talairach or Montreal Neurologic Institute (MNI) information. We only selected articles in English. Literature such as reviews and meta-analysis articles were excluded. Articles with incomplete information or secondary processing were also excluded.

#### Data Extraction

Two researchers independently selected, extracted, and checked the data. When there is any disagreement, a third reviewer participated in the decision. All of the abnormal brain region coordinates were derived from the consistent text.

### Data Analysis Program

We identified both increased and decreased indicators. The ALFF/fALFF ratio was increased in 32 foci (*n* = 169) and decreased in 11 foci (*n* = 202); ReHo was increased in 6 foci (*n* = 95) and decreased in 7 foci (*n* = 95); and FC was increased in 24 foci (*n* = 122) and decreased in 44 foci (*n* = 136).

We used a Java-based version of Ginger ALE 2.3.6 (http://www.brainmap.org/ale) to perform the ALE meta-analysis. The convergence of the difference in foci was assessed between the sVCI and HC groups. The steps were as follows: (1) import a text file used to read the data into the software; (2) set a threshold at *p* < 0.05; (3) input the maps into the MNI template and view the image with dpabi software (http://fmri.org/dpabi).

## Results

### Research Results

The study characteristics and results are summarized in Table 1.

**Table 1:** Demographic data and clinical information.

**Study**	**G**	***N***	**Age (SD)**	**Gender (male/female)**	**MMSE (SD)**	**Group contrasts**	**Foci**	**Correction for multiple comparisons**
**ALFF**								
(19)	sVCI	11	40.2 (11.2)	4/7	19.6 (4.3)	sVCI>HC	15	*p* < 0.05 (cor)
	HC	22	40.2 (87.2)	10/12	28.5 (1.5)	sVCI<HC	3	
(20)	sVCI	30	69.0 (7.8)	19/11	68.0 (5.8)	sVCI>HC	6	*p* < 0.01 (cor)
	HC	35	68.0 (5.8)	22/13	28.4 (1.1)	sVCI<HC	2	
(21)	sVCI	30	69.0 (7.8)	19/11	16.1 (5.1)	sVCI>HC	3	*p* < 0.01 (cor)
	HC	35	68.0 (5.8)	22/13	28.4 (1.1)	sVCI<HC	1	
(22)	sVCI	22	79 (6)	16/6	25 (2.05)	sVCI>HC	2	*p* < 0.05(cor)
	HC	28	70 (9)	17/11	29 (1.09)	sVCI<HC	2	
(23)	sVCI	22	49.0 (14.2)	13/9	23.3 (6.3)	sVCI>HC	3	*p* < 0.05 (cor)
	HC	44	48.5 (13.7)	26/18	28.6 (1.1)	sVCI<HC	1	
(24)	sVCI	46	59.99 (8.59)	22/24	23.23 (2.70)	sVCI>HC	1	*p* < 0.05 (cor)
	HC	28	58.35 (6.82)	13/15	29.46 (1.07)	sVCI<HC	1	
(25)	sVCI	28	65.3 (8.1)	12/16	29.1 (1.2)	sVCI>HC	2	*p* < 0.01 (cor)
	HC	26	66.7 (9.5)	11/15	25.7 (2.7)	sVCI<HC	1	
**FC**								
(26)	sVCI	32	70.09 (8.26)	14/18	23.78 (2.66)	sVCI>HC	2	*p* < 0.05 (cor)
	HC	23	68.87 (7.05)	14/9	27.96 (0.98)	sVCI<HC	8	
(27)	sVCI	22	48.9 (14.2)	13/9	23.5 (5.7)	sVCI>HC	3	*p* < 0.05 (cor)
	HC	44	48.4 (13.7)	26/18	28.2 (1.3)	sVCI<HC	6	
(28)	sVCI	29	71.07 (6.72)	19/10	25.90 (3.09)	sVCI>HC	3	*p* < 0.05 (cor)
	HC	22	67.78 (6.75)	14/8	28.61 (1.23)	sVCI<HC	7	
(29)	sVCI	16	69.1 (7.8)	14/2	28.1 (1.4)	sVCI>HC	5	*p* < 0.01 (cor)
	HC	18	66.2 (7.7)	16/2	28.9 (1.3)	sVCI<HC	5	
(30)	SVCI	54	70.48 (4.81)	32/22	25.80 (2.48)	sVCI>HC	6	*p* < 0.05 (cor)
	NC	27	67.63 (8.19)	10/17	27.93 (1.03)	sVCI<HC	5	
(23)	sVCI	22	49.0 (14.2)	13/9	23.3 (6.3)	sVCI>HC	0	*p* < 0.05 (cor)
	HC	44	48.5 (13.7)	26/18	28.6 (1.1)	sVCI<HC	8	
(31)	sVCI	14	66.00 (5.13)	7/7	26.86 (2.66)	sVCI>HC	5	
	HC	33	62.03 (7.53)	16/17	28.47 (1.49)	sVCI<HC	0	
(32)	sVCI	31	63.84 (14.1)	18/13	26.32 (2.06)	sVCI>HC	0	*p* < 0.05 (cor)
	HC	32	62.72 (8.22)	18/14	28.75 (1.39)	sVCI<HC	5	
**ReHo**								
(32)	sVCI	31	63.84 (14.1)	18/13	26.32 (2.06)	sVCI>HC	0	*p* < 0.05 (cor)
	HC	32	62.72 (8.22)	18/14	28.75 (1.39)	sVCI<HC	2	
(31)	sVCI	14	66.00 (5.13)	7/7	26.86 (2.66)	sVCI>HC	3	*p* < 0.05 (cor)
	HC	33	62.03 (7.53)	16/17	28.47 (1.49)	sVCI<HC	0	
(33)	SIVD	20	75.8 (7.67)	13/7	20.1 (5.88)	sVCI>HC	2	*p* < 0.05 (cor)
	HC	23	65.1 (6.97)	11/12	27.9 (1.60)	sVCI<HC	1	
(22)	sVCI	22	79 (6)	16/6	25 (2.05)	sVCI>HC	1	*p* < 0.05 (cor)
	HC	28	70 (9)	17/11	29 (1.09)	sVCI<HC	3	
(27)	sVCI	22	48.9 (14.2)	13/9	23.5 (5.7)	sVCI>HC	0	*p* < 0.05(cor)
	HC	44	48.4 (13.7)	26/18	28.2 (1.3)	sVCI<HC	1	

### Meta-Analysis Results

**Figure 2:**
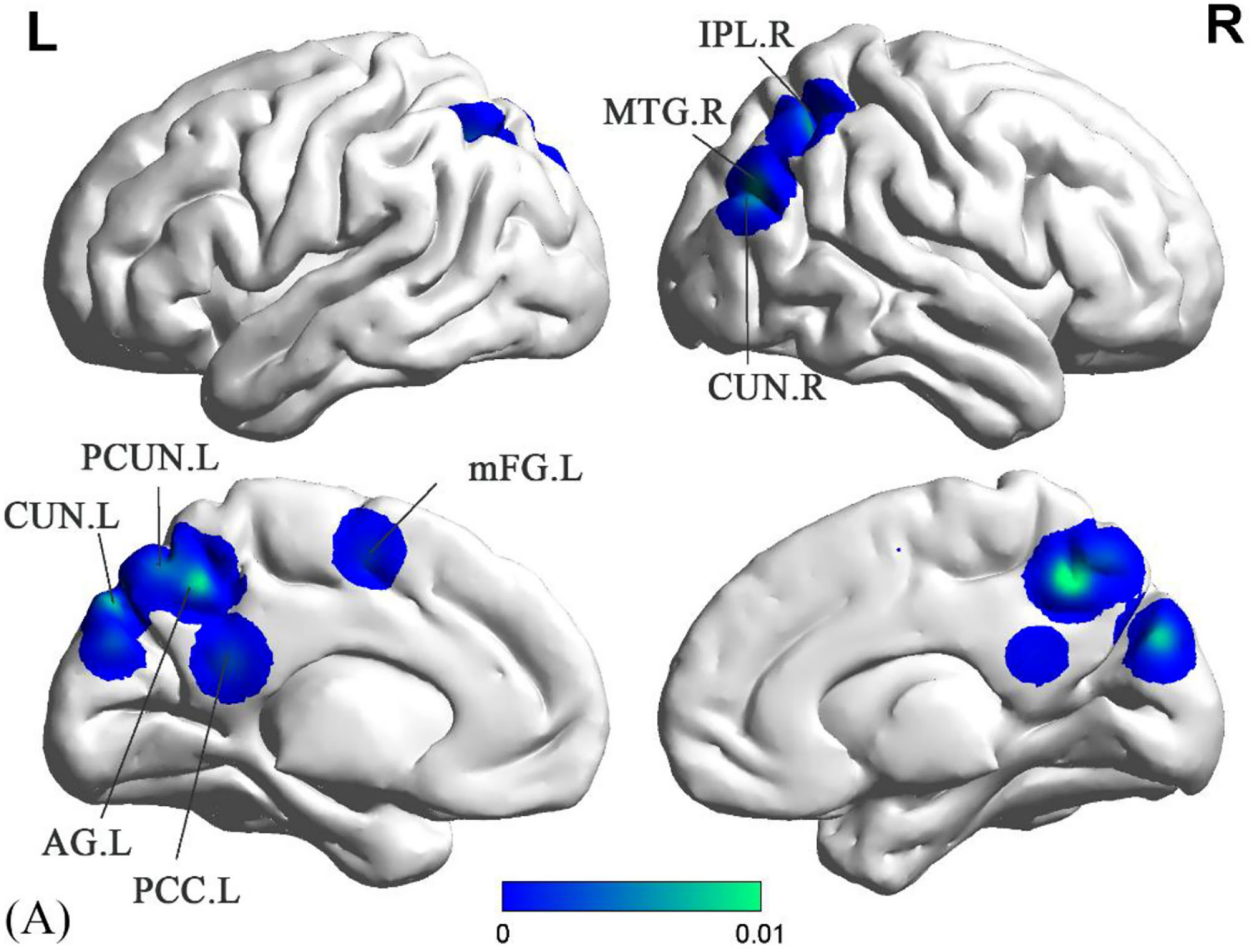
Decreased ALFF in sVCI patients compared with HCs. sVCI, subcortical cognitive impairment; HCs, healthy controls; ALFF, amplitude of low-frequency fluctuation; PCUN, precuneus; PCC, posterior cingulate cortex; CUN, cuneus; MTG, middle temporal gyrus; IPL, inferior parietal lobule; AG, angular gyrus; Mfg, medial frontal gyrus; R, right; L, left.

Compared to the HC groups, sVCI patients had no increased ALFF in the specific brain regions. In contrast, the brain regions with a significant decrease in ALFF values mainly included the precuneus (PCUN), posterior cingulate cortex (PCC), cuneus (CUN), middle temporal gyrus (MTG), inferior parietal lobule (IPL), angular gyrus (AG), and the medial frontal gyrus (mFG) (Figure 2 and Table 2).

**Table 2:** All brain regions and clusters derived from meta-analysis.

**Cluster**	**Volume (mm^**3**^)**	**MNI**			**Anatomical regions**	**Maximum ALE value**	**Side**	**BA**
		**X**	**Y**	**Z**				
**ALFF**								
**sVCI>HC**								
None								
**sVCI < HC**								
1	44,904	2	−54	48	Precuneus	0.017697	Left	7
1	44,904	−6	−45	24	Posterior cingulate cortex	0.008758	Left	30
1	44,904	6	−81	30	Cuneus	0.008326	Right	18
1	44,904	−8	−80	42	Cuneus	0.008056	Left	19
1	44,904	0	−66	54	Precuneus	0.006929	Left	7
2	17,440	48	−72	30	Middle temporal gyrus	0.00918	Right	39
2	17,440	42	−54	50	Inferior parietal lobule	0.006901	Right	40
3	11,360	−30	−60	44	Angular gyrus	0.006901	Left	39
4	9,664	−9	−3	57	Medial frontal gyrus	0.007794	Left	6
**ReHo**								
**sVCI>HC**								
1	30,608	24	−68	36	Precuneus	0.007556	Right	7
1	30,608	42	−51	39	Inferior parietal lobule	0.007112	Right	40
2	15,872	−18	−60	51	Cingulate gyrus	0.007331	Left	7
3	14,400	−12	21	30	Precuneus	0.008136	Left	32
4	13,576	53	−31	−23	Fusiform gyrus	0.007794	Right	20
5	12,872	39	51	15	Middle frontal gyrus	0.007624	Right	10
**sVCI < HC**								
1	32,920	2	−76	30	Precuneus	0.011713	Left	31
1	32,920	15	−87	6	Lingual gyrus	0.008056	Right	17
2	14,400	−39	0	12	Insula	0.008326	Left	13
3	13,216	−21	−78	−33	Pyramis	0.008618	Left	-
4	13,216	21	3	6	Lentiform nucleus	0.008618	Right	-
5	12,872	39	51	15	Middle frontal gyrus	0.007624	Right	10
**FC**								
**sVCI>HC**								
1	19,800	40	6	28	Precentral gyrus	0.00901	Right	6
1	19,800	48	44	24	Superior frontal gyrus	0.008695	Right	9
1	19,800	50	24	22	Middle frontal gyrus	0.008292	Right	6
1	19,800	28	50	24	Superior frontal gyrus	0.007415	Right	9
**sVCI < HC**								
1	14,976	−34	−6	42	Middle frontal gyrus	0.008927	Left	6
1	14,976	−36	−18	50	Postcentral gyrus	0.008331	Left	3
1	14,976	−50	−20	38	Postcentral gyrus	0.007795	Left	2
1	14,976	−36	0	28	Precentral gyrus	0.007652	Left	6
2	13,800	48	6	48	Middle frontal gyrus	0.009673	Left	6
2	13,800	42	14	30	Precentral gyrus	0.009003	Right	9
2	13,800	48	8	40	Middle frontal gyrus	0.008929	Right	6
2	13,800	64	2	12	Precentral gyrus	0.008695	Right	6
2	13,800	48	4	22	Precentral gyrus	0.00806	Right	6
4	8,736	22	28	36	Sub-gyral	0.008695	Right	8
4	8,736	10	36	42	Medial frontal gyrus	0.008695	Right	8
4	8,736	32	38	30	Middle frontal gyrus	0.008621	Right	9

*BA, Brodmann Area; ALE, Anatomical/Activation Likelihood Estimation; MNI, Montreal Neurologic Institute; sVCI, subcortical vascular cognitive impairment; HCs, healthy controls; ALFF, the amplitude of low-frequency fluctuation; ReHo, regional homogeneity; FC, functional connectivity*.

**Figure 3:**
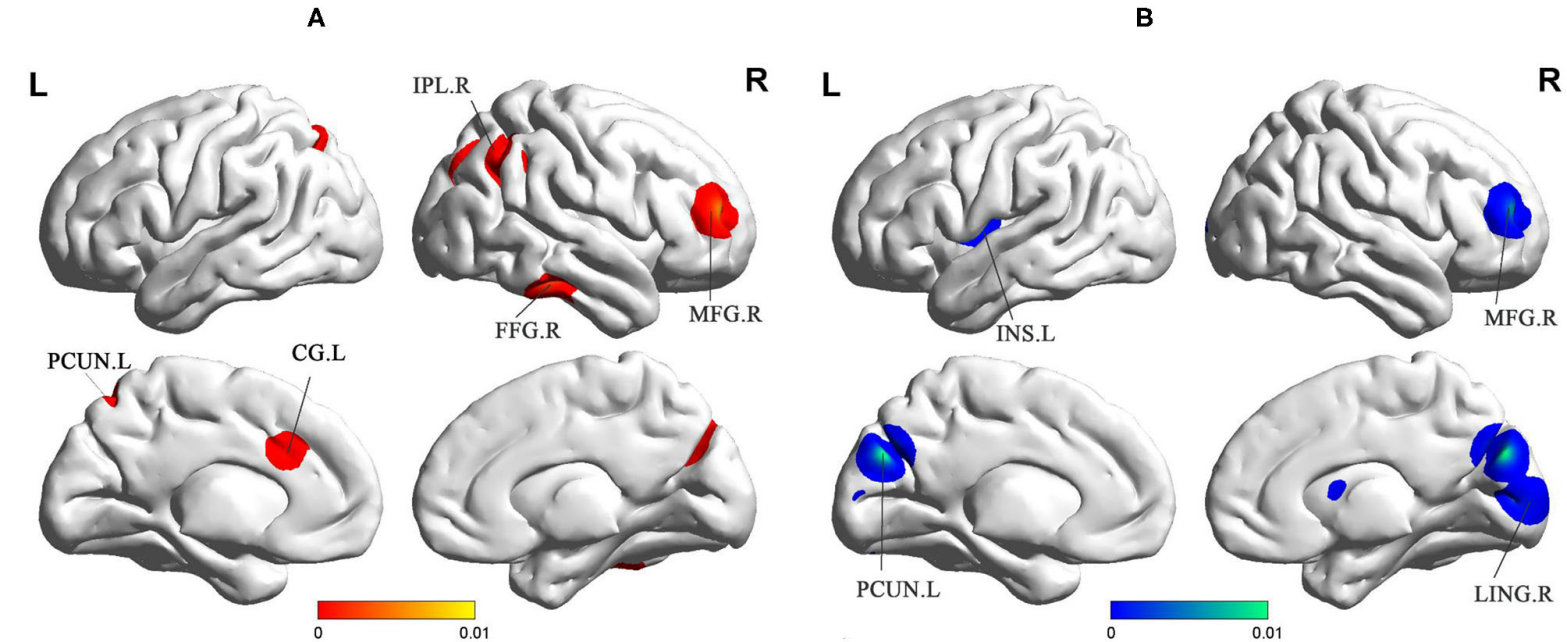
**(A)** Increased ReHo in sVCI patients compared with HCs. **(B)** Decreased ReHo in sVCI patients compared with HCs. sVCI, subcortical cognitive impairment; HCs, healthy controls; ReHo, regional homogeneity; PCUN, precuneus; IPL, inferior parietal lobule; CG, cingulate gyrus; INS, insula; FFG, fusiform gyrus; MFG, middle frontal gyrus; LING, lingual gyrus; R, right; L, left.

sVCI patients had increased ReHo in the PCUN, IPL, cingulate gyrus (CG), fusiform gyrus (FFG), and middle frontal gyrus (MFG) (Figure 3 and Table 2). In addition, sVCI patients presented with decreased ReHo in the PCUN, lingual gyrus (LING), insula, posterior lobe, lentiform nucleus, and MFG (Figure 3 and Table 2).

**Figure 4:**
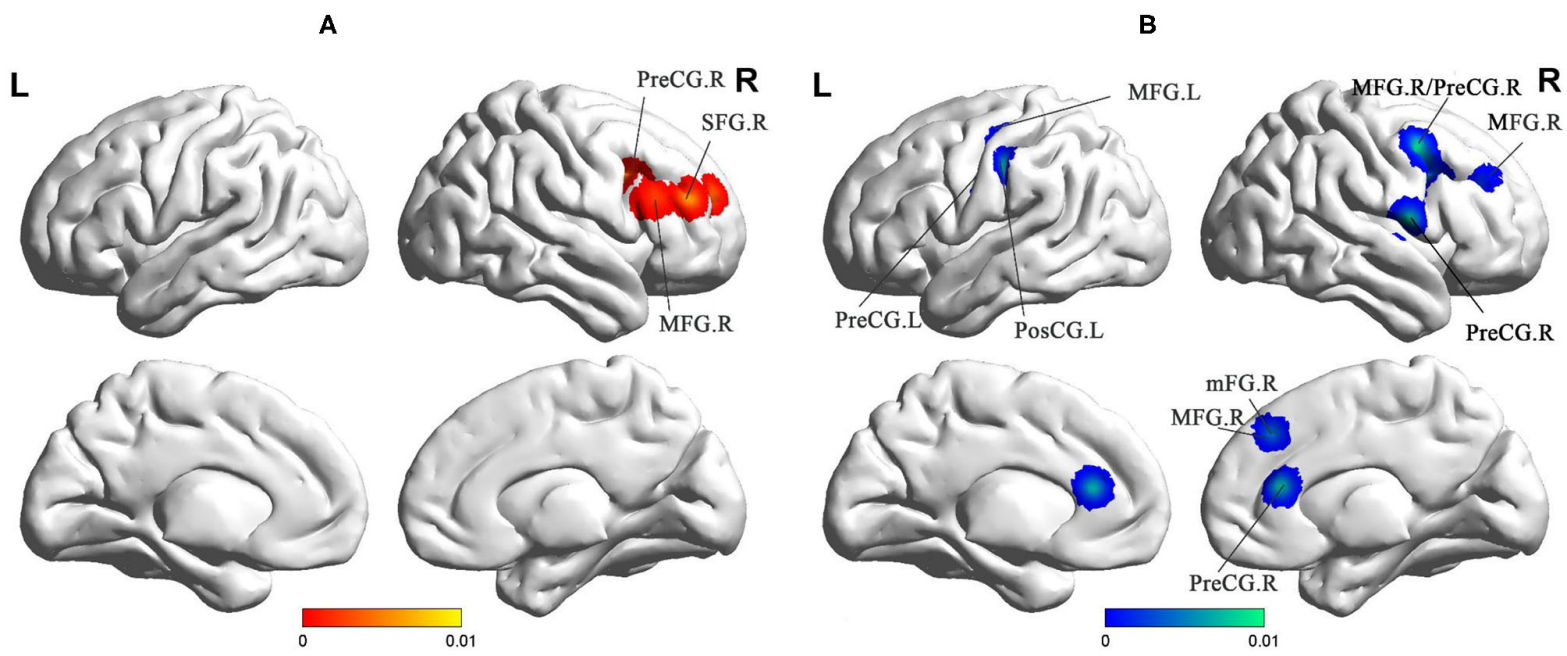
**(A)** Increased FC in sVCI patients compared with HCs. **(B)** Increased FC in sVCI patients compared with HCs. sVCI, subcortical cognitive impairment; HCs, healthy controls; FC, functional connectivity; PreCG, precentral gyrus; SFG, superior frontal gyrus; MFG, middle frontal gyrus; PosCG, postcentral gyrus; mFG, medial frontal gyrus; MFG, middle frontal gyrus; R, right; L, left.

Compared to the HC groups, sVCI patients showed increased FC in the precentral gyrus (PreCG), superior frontal gyrus (SFG), and MFG (Figure 4 and Table 2). sVCI patients showed decreased FC in the MFG, postcentral gyrus (PosCG), PreCG, sub-gyral, mFG, and MFG (Figure 4 and Table 2).

## Discussion

The present study was the first meta-analysis conducted to explore the specific functional alterations in brain regions in sVCI patients. We determined different patterns of spontaneous brain activity through two indicators, ALFF and ReHo, and found different brain functional connections by comparing the results. In patients with sVCI, the areas most affected in the brain were the frontal lobes, parietal lobes, and occipital lobes. Additionally, the significant difference in the limbic system may indicate spatially distinct patterns of brain function in sVCI patients.

Decreases in ALFF were found in the PCUN, PCC, MTG, IPL, and AG. ReHo was also decreased in the PCUN and increased in the PCUN and IPL. As the medial aspect of the parietal lobe, the PCUN is considered the hub of multiple brain networks (34). The PCUN has three separate areas that participate in different functional networks (35). The anterior PCUN mainly links to sensorimotor areas and projects into the insula. The central PCUN is related to the hippocampus and the posterior PCUN joins with regions of the visual cortex (35). Previous evidence reported in a positron emission tomography (PET) study showed hypometabolism of the PCUN in sVCI patients (36). It is known that the IPL is responsible for maintaining attention control and dealing with information (37). The AG is involved in language, number processing, and memory retrieval (38). As an important part of the limbic system, the PCC has extensive functional connections with many brain areas in the prefrontal lobe. Weak signal values in this region, which is responsible for monitoring sensation, stereotyping, and memory, suggest that cognitive impairment in sVCI patients is closely related to this important brain region (39). A recent study revealed that the PCC and PCUN appeared to have the most structural and functional alterations in sVCI patients (40). Some studies reported that the language understanding network, centered on the dominant hemisphere of the MTG, is the neural basis of the brain's language understanding function (41). As we know, language impairment is an important manifestation of vascular dementia (42).

All of the brain regions mentioned above are part of the DMN, the main function of which is to extract the process of episodic memory, cognition, and emotion (43). As for both rising and falling signals in the same brain region, this is due to different stages of the disease (44). It has been shown that during the development of sVCI, some brain regions are compensated for to coordinate cognitive functions (45). Thus, it is not hard to explain why there are elevated signals in certain brain regions. In summary, brain function damage caused by sVCI was concentrated in the DMN, which is consistent with the impairment of episodic memory shown in sVCI patients. However, the within-group analysis showed no significant differences in the increased ALFF values.

Brain regions such as the MFG, SFG, mFG, and PreCG exhibited abnormal FC. These brain regions were located in the ECN, which aims at integrating sensory and memory information and regulating cognition and behavior (30). The ECN, whose core is the prefrontal lobe, is in charge of the integration of sensation, memory information, and the regulation of cognition and behavior (46). Executive function has been confirmed to be the main cognitive domain that shows degeneration in sVCI (29). A structural equation model on CSVD showed that the main neuropsychological symptom, executive dysfunction, may predict both reductions in awareness and the quality of life (47). The pathogenesis of sVCI is mainly the long-term damage of gray matter and white matter. Naturally, the course of the disease shows a trend of gradual aggravation. As a result, it is significant to conclude that the involved brain regions were located in the ECN based on the existing literature. Recent findings pointed out that the employment of transcranial magnetic stimulation could promote an increase in global excitability in sVCI (42). Our research can provide meaningful potential targets for transcranial magnetic stimulation (TMS) to enhance cortical excitability and synaptic plasticity.

Our study showed decreased ALFF in the cuneus and decreased ReHo in the LING. Located at the center of the visual cortex, the LING and the cuneus are in charge of visual function processes (48). A randomized controlled trial confirmed that visual dysfunction may exist in sVCI and regular ophthalmological exams can help improve the quality of life (49). Our findings were consistent with the clinical symptoms. There was a synergistic effect between the networks. When the DMN and ECN are abnormal, the VN will also be functionally damaged (49). As for the compensatory increase in the VN in the later period, more experiments are needed to prove this. Regardless, ReHo tends to demonstrate the coherence of neural activity. Interestingly, a decrease in ReHo in the insula was a unique and important discovery in sVCI patients (50). Covered by the frontal and temporal opercula, the insula contains several functional regions involved in attention, language, speech, and working memory. It is also the hub of several important networks (51). As mentioned in the DMN above, the insula is connected to the anterior PCUN, both of which are reduced. Thus, brain regions and brain networks interact with each other in sVCI patients (49).

## Limitations

Although the results of this study are valuable, limitations were inevitable. The heterogeneity of the data sources is clear. Different threshold settings and preprocessing methods used in the studies would affect our results to different extents (52). However, these differences were negligible with respect to the neuroimaging changes. Since CSVD is a broad concept that includes a wide range of diseases, there were limitations in this area even though the pathogenesis was similar. Due to the small number of studies, it was difficult to study the detailed unified classification. Another limitation was confounding factors such as load of white matter lesions, the presence of microbleeds, and infarcts, which influenced the resting-state indexes to some degree. Besides, as the domains of cognitive impairment were different in the included papers, the heterogeneity of the article was undeniable. Finally, because it is difficult to get the full text of several literatures, this is also the limitation of this paper.

## Clinical Implications

Although a single study can provide valuable information, it still lacks uniformity and precision. Considering this, our quantitative analysis was very necessary. Core brain areas such as the PCUN, LING, frontal gyrus, and insula were regarded as neuroimaging markers in sVCI. The findings also provide a valuable basis for TMS and drug treatment. Having identified which functions were impaired, we can follow up regularly, such as focusing on visual function, to improve the patients' quality of life. In conclusion, our results showed disease-specific brain area damage characteristics and provide information for follow-up care and treatment.

## Conclusion

By synthesizing the published literature on sVCI caused by cerebral microvascular disease, we obtained special imaging markers, which were concentrated on the frontal lobes including the middle frontal gyrus, superior frontal gyrus, medial frontal gyrus, precentral gyrus; parietal lobes including the precuneus, angular gyrus, postcentral gyrus, and inferior parietal lobule; occipital lobes including the lingual gyrus and cuneus; temporal lobes including the fusiform gyrus and middle temporal gyrus; and the limbic system including the cingulate gyrus. By dividing these brain regions, the brain networks to which they belonged were also identified. Multiple brain functional networks such as the DMN, ECN, and VN showed abnormal performance consistent with sVCI patients. These findings objectively present the exact abnormalities within the brain, which can help to further understand the pathogenesis of sVCI and suggest imaging findings as a potential biomarker. It may also provide a basis for new treatment approaches.

## Data Availability Statement

The original contributions presented in the study are included in the article/Supplementary Materials, further inquiries can be directed to the corresponding author.

## Author Contributions

JC and XL designed the study and revised it critically for important content. WX performed the research and drafted the manuscript. YS and SC helped in data analyses. CX, GH, and WM helped in clinical data collection and analyses.

## Funding

This study was supported by the National Natural Science Foundation of China (No. 81701675); the Key Project supported by the Medical Science and Technology Development Foundation, Nanjing Department of Health (No. JQX18005); the Cooperative Research Project of Southeast University-Nanjing Medical University (No. 2018DN0031); the Key Research and Development Plan (Social Development) Project of Jiangsu Province (No. BE2018608); and the Innovation and Entrepreneurship Training Program for College Students in Jiangsu Province (Nos. 201810312061X and 201910312035Z).

## Conflict of Interest

The authors declare that the research was conducted in the absence of any commercial or financial relationships that could be construed as a potential conflict of interest.

## Publisher's Note

All claims expressed in this article are solely those of the authors and do not necessarily represent those of their affiliated organizations, or those of the publisher, the editors and the reviewers. Any product that may be evaluated in this article, or claim that may be made by its manufacturer, is not guaranteed or endorsed by the publisher.

## References

[1] Pasi M Cordonnier C. Clinical relevance of cerebral small vessel diseases. Stroke. (2020) 51:47–53. 10.1161/STROKEAHA.119.024148 31752613

[2] Gorelick PB Counts SE Nyenhuis D. Vascular cognitive impairment and dementia. Biochim Biophys Acta. (2016) 1862:860–8. 10.1016/j.bbadis.2015.12.015 26704177 PMC5232167

[3] Jang H Kim HJ Park S Park YH Choe Y Cho H. Application of an amyloid and tau classification system in subcortical vascular cognitive impairment patients. Eur J Nuclear Med Mol Imaging. (2020) 47:292–303. 10.1007/s00259-019-04498-y 31471715

[4] Sun Y Cao W Ding W Wang Y Han X Zhou Y. Cerebral blood flow alterations as assessed by 3D ASL in cognitive impairment in patients with subcortical vascular cognitive impairment: a marker for disease severity. Front Aging Neurosci. (2016) 8:211. 10.3389/fnagi.2016.00211 27630562 PMC5005930

[5] Kalaria RN Akinyemi R Ihara M. Stroke injury, cognitive impairment and vascular dementia. Biochim Biophys Acta. (2016) 1862:915–25. 10.1016/j.bbadis.2016.01.015 26806700 PMC4827373

[6] Yang Y Kimura-Ohba S Thompson J Rosenberg GA. Rodent models of vascular cognitive impairment. Transl Stroke Res. (2016) 7:407–14. 10.1007/s12975-016-0486-2 27498679 PMC5016244

[7] Müller K Courtois G Ursini MV Schwaninger M. New insight into the pathogenesis of cerebral small-vessel diseases. Stroke. (2017) 48:520–7. 10.1161/STROKEAHA.116.012888 28082670

[8] Zhu S Nahas SJ. CADASIL: imaging characteristics and clinical correlation. Curr Pain Headache Rep. (2016) 20:57. 10.1007/s11916-016-0584-6 27591799

[9] Smitha KA Akhil Raja K Arun KM Rajesh PG Thomas B Kapilamoorthy TR. Resting state fMRI: a review on methods in resting state connectivity analysis and resting state networks. Neuroradiol J. (2017) 30:305–17. 10.1177/1971400917697342 28353416 PMC5524274

[10] Wang JJ Chen X Sah SK Zeng C Li YM Li N. Amplitude of low-frequency fluctuation (ALFF) and fractional ALFF in migraine patients: a resting-state functional MRI study. Clin Radiol. (2016) 71:558–64. 10.1016/j.crad.2016.03.004 27055741

[11] Zang Y Jiang T Lu Y He Y Tian L. Regional homogeneity approach to fMRI data analysis. NeuroImage. (2004) 22:394–400. 10.1016/j.neuroimage.2003.12.030 15110032

[12] Shen X Wu J Wang Z Chen T. Characterization of in vitro neural functional connectivity on a neurofluidic device. Electrophoresis. (2019) 40:2996–3004. 10.1002/elps.201900168 31556965

[13] Zhu Y Gong L He C Wang Q Ren Q Xie C. Default mode network connectivity moderates the relationship between the APOE genotype and cognition and individualizes identification across the Alzheimer's disease spectrum. J Alzheimers Dis. (2019) 70:843–60. 10.3233/JAD-190254 31282419

[14] DeRamus TP Kana RK. Anatomical likelihood estimation meta-analysis of grey and white matter anomalies in autism spectrum disorders. Neuroimage Clin. (2015) 7:525–36. 10.1016/j.nicl.2014.11.004 25844306 PMC4375647

[15] Fornito A Yücel M Patti J Wood SJ Pantelis C. Mapping grey matter reductions in schizophrenia: an anatomical likelihood estimation analysis of voxel-based morphometry studies. Schizophr Res. (2009) 108:104–13. 10.1016/j.schres.2008.12.011 19157788

[16] Robinson JL Laird AR Glahn DC Blangero J Sanghera MK Pessoa L. The functional connectivity of the human caudate: an application of meta-analytic connectivity modeling with behavioral filtering. NeuroImage. (2012) 60:117–29. 10.1016/j.neuroimage.2011.12.010 22197743 PMC3288226

[17] Li Q Yang Y Reis C Tao T Li W Li X. Cerebral small vessel disease. Cell Transpl. (2018) 27:1711–22. 10.1177/0963689718795148 30251566 PMC6300773

[18] Teng Z Dong Y Zhang D An J Lv P. Cerebral small vessel disease and post-stroke cognitive impairment. Int J Neurosci. (2017) 127:824–30. 10.1080/00207454.2016.1261291 27838946

[19] Lei Y Li Y Ni W Jiang H Yang Z Guo Q. Spontaneous brain activity in adult patients with moyamoya disease: a resting-state fMRI study. Brain Res. (2014) 1546:27–33. 10.1016/j.brainres.2013.12.022 24380677

[20] Li C Liu C Yin X Yang J Gui L Wei L. Frequency-dependent changes in the amplitude of low-frequency fluctuations in subcortical ischemic vascular disease (SIVD): a resting-state fMRI study. Behav Brain Res. (2014) 274:205–10. 10.1016/j.bbr.2014.08.019 25138697

[21] Liu C Li C Yin X Yang J Zhou D Gui L. Abnormal intrinsic brain activity patterns in patients with subcortical ischemic vascular dementia. PLoS ONE. (2014) 9:e87880. 10.1371/journal.pone.0087880 24498389 PMC3912127

[22] Ni L Liu R Yin Z Zhao H Nedelska Z Hort J. Aberrant spontaneous brain activity in patients with mild cognitive impairment and concomitant lacunar infarction: a resting-state functional mri study. J Alzheimers Dis. (2016) 50:1243–54. 10.3233/JAD-150622 26836013

[23] Su J Wang M Ban S Wang L Cheng X Hua F. Relationship between changes in resting-state spontaneous brain activity and cognitive impairment in patients with CADASIL. J Headache Pain. (2019) 20:36. 10.1186/s10194-019-0982-3 30995925 PMC6734224

[24] Wang J Chen H Liang H Wang W Liang Y Liang Y. Low-frequency fluctuations amplitude signals exhibit abnormalities of intrinsic brain activities and reflect cognitive impairment in leukoaraiosis patients. Med Sci Monit. (2019) 25:5219–28. 10.12659/MSM.915528 31302662 PMC6650186

[25] Yi L Wang J Jia L Zhao Z Lu J Li K. Structural and functional changes in subcortical vascular mild cognitive impairment: a combined voxel-based morphometry and resting-state fMRI study. PLoS ONE. (2012) 7:e44758. 10.1371/journal.pone.0044758 23028606 PMC3446994

[26] Zhou X Hu X Zhang C Wang H Zhu X Xu L. Aberrant functional connectivity and structural atrophy in subcortical vascular cognitive impairment: relationship with cognitive impairments. Front Aging Neurosci. (2016) 8:14. 10.3389/fnagi.2016.00014 26869922 PMC4736471

[27] Su J Ban S Wang M Hua F Wang L Cheng X. Reduced resting-state brain functional network connectivity and poor regional homogeneity in patients with CADASIL. J Headache Pain. (2019) 20:103. 10.1186/s10194-019-1052-6 31711415 PMC6849263

[28] Ding W Cao W Wang Y Sun Y Chen X Zhou Y. Altered functional connectivity in patients with subcortical vascular cognitive impairment–a resting-state functional magnetic resonance imaging study. PLoS ONE. (2015) 10:e0138180. 10.1371/journal.pone.0138180 26376180 PMC4573963

[29] Sun YW Qin LD Zhou Y Xu Q Qian LJ Tao J. Abnormal functional connectivity in patients with vascular cognitive impairment, no dementia: a resting-state functional magnetic resonance imaging study. Behav Brain Res. (2011) 223:388–94. 10.1016/j.bbr.2011.05.006 21605598

[30] Liu X Chen L Cheng R Luo T Lv F Fang W. Altered functional connectivity in patients with subcortical ischemic vascular disease: a resting-state fMRI study. Brain Res. (2019) 1715:126–33. 10.1016/j.brainres.2019.03.022 30910630

[31] Ye Q Chen X Qin R Huang L Yang D Liu R. Enhanced regional homogeneity and functional connectivity in subjects with white matter hyperintensities and cognitive impairment. Front Neurosci. (2019) 13:695. 10.3389/fnins.2019.00695 31333409 PMC6617843

[32] Zuo M Xu Y Zhang X Li M Jia X Niu J. Aberrant brain regional homogeneity and functional connectivity of entorhinal cortex in vascular mild cognitive impairment: a resting-state functional MRI study. Front Neurol. (2018) 9:1177. 10.3389/fneur.2018.01177 30723453 PMC6350268

[33] Tu MC Hsu YH Yang JJ Huang WH Deng JF Lin SY. Attention and functional connectivity among patients with early-stage subcortical ischemic vascular disease and Alzheimer's disease. Front Aging Neurosci. (2020) 12:239. 10.3389/fnagi.2020.00239 32903858 PMC7439096

[34] Cunningham SI Tomasi D Volkow ND. Structural and functional connectivity of the precuneus and thalamus to the default mode network. Hum Brain Mapping. (2017) 38:938–56. 10.1002/hbm.23429 27739612 PMC6866740

[35] Wang Z Fei L Sun Y Li J Wang F Lu Z. The role of the precuneus and posterior cingulate cortex in the neural routes to action. Comput Assist Surg (Abingdon). (2019) 24:113–20. 10.1080/24699322.2018.1557903 30607999

[36] Kato T Inui Y Nakamura A Ito K. Brain fluorodeoxyglucose (FDG) PET in dementia. Ageing Res Rev. (2016) 30:73–84. 10.1016/j.arr.2016.02.003 26876244

[37] Igelström KM Graziano MSA. The inferior parietal lobule and temporoparietal junction: a network perspective. Neuropsychologia. (2017) 105:70–83. 10.1016/j.neuropsychologia.2017.01.001 28057458

[38] Seghier ML. The angular gyrus: multiple functions and multiple subdivisions. Neuroscientist. (2013) 19:43–61. 10.1177/1073858412440596 22547530 PMC4107834

[39] Fu Z Caprihan A Chen J Du Y Adair JC Sui J. Altered static and dynamic functional network connectivity in Alzheimer's disease and subcortical ischemic vascular disease: shared and specific brain connectivity abnormalities. Hum Brain Mapp. (2019) 40:3203–21. 10.1002/hbm.24591 30950567 PMC6865624

[40] Noh Y Seo SW Jeon S Lee JM Kim JS Lee JH. The role of cerebrovascular disease in amyloid deposition. J Alzheimers Dis. (2016) 54:1015–26. 10.3233/JAD-150832 27567803

[41] Jabbour RA Penovich PE Risse GL Kispert DE Dunn MB Gates JR. Atypical language cortex in the left temporal lobe: relationship to bilateral language. Neurology. (2004) 63:1833–7. 10.1212/01.WNL.0000144273.82654.96 15557498

[42] Cantone M Lanza G Fisicaro F Pennisi M Bella R Di Lazzaro V. Evaluation and treatment of vascular cognitive impairment by transcranial magnetic stimulation. Neural Plasticity. (2020) 2020:8820881. 10.1155/2020/8820881 33193753 PMC7641667

[43] Yuan B Chen J Gong L Shu H Liao W Wang Z. Mediation of episodic memory performance by the executive function network in patients with amnestic mild cognitive impairment: a resting-state functional MRI study. Oncotarget. (2016) 7:64711–25. 10.18632/oncotarget.11775 27589839 PMC5323110

[44] Simó M Rifà-Ros X Vaquero L Ripollés P Cayuela N Jové J. Brain functional connectivity in lung cancer population: an exploratory study. Brain Imaging Behav. (2018) 12:369–82. 10.1007/s11682-017-9697-8 28290076

[45] Liu Y Chen Y Liang X Li D Zheng Y Zhang H. Altered resting-state functional connectivity of multiple networks and disrupted correlation with executive function in major depressive disorder. Front Neurol. (2020) 11:272. 10.3389/fneur.2020.00272 32411071 PMC7198729

[46] Hojjati SH Ebrahimzadeh A Khazaee A Babajani-Feremi A. Predicting conversion from MCI to AD by integrating rs-fMRI and structural MRI. Comput Biol Med. (2018) 102:30–9. 10.1016/j.compbiomed.2018.09.004 30245275

[47] Brookes RL Herbert V Paul S Hannesdottir K Markus HS Morris RG. Executive dysfunction, awareness deficits and quality of life in patients with cerebral small vessel disease: a structural equation model. Neuropsychology. (2014) 28:247–53. 10.1037/neu0000015 24274025

[48] Li S Lv P He M Zhang W Liu J Gong Y. Cerebral regional and network characteristics in asthma patients: a resting-state fMRI study. Front Med. (2020) 14:792–801. 10.1007/s11684-020-0745-1 32270434

[49] Kidoh M Utsunomiya D Funama Y Ashikaga H Nakaura T Oda S. Vectors through a cross-sectional image (VCI): a visualization method for four-dimensional motion analysis for cardiac computed tomography. J Cardiovasc Comput Tomogr. (2017) 11:468–73. 10.1016/j.jcct.2017.09.010 28967574 PMC5712277

[50] Xu X Li W Mei J Tao M Wang X Zhao Q. Feature selection and combination of information in the functional brain connectome for discrimination of mild cognitive impairment and analyses of altered brain patterns. Front Aging Neurosci. (2020) 12:28. 10.3389/fnagi.2020.00028 32140102 PMC7042199

[51] Gasquoine PG. Contributions of the insula to cognition and emotion. Neuropsychol Rev. (2014) 24:77–87. 10.1007/s11065-014-9246-9 24442602

[52] Pardoe H Pell GS Abbott DF Berg AT Jackson GD. Multi-site voxel-based morphometry: methods and a feasibility demonstration with childhood absence epilepsy. Neuroimage. (2008) 42:611–6. 10.1016/j.neuroimage.2008.05.007 18585930 PMC2603188

