# Supplementary material for: An ALE Meta-Analysis of Specific Functional MRI Studies on Subcortical Vascular Cognitive Impairment
Source: Front Neurol. 2021 Sep 22;12:649233. doi: 10.3389/fneur.2021.649233 (PMC8492914; doi:10.3389/fneur.2021.649233)
Supplement: Supplementary file 1 [file Table_1.DOCX]

**Supplementary Table 1** Search terms

| **Database** | **Search terms** |
| --- | --- |
| PubMed | All Fields: 1) (“functional magnetic resonance imaging [MeSH] OR “RESTING STATE” [MeSH]) AND (“subcortical vascular cognitive impairment”[MeSH]) AND (“Functional connectivity”) ; 2) (“functional magnetic resonance imaging [MeSH] OR “RESTING STATE” [MeSH]) AND (“subcortical vascular cognitive impairment”[MeSH] AND “regional homogeneity”); 3) (“functional magnetic resonance imaging [MeSH] OR “RESTING STATE” [MeSH]) AND (“subcortical vascular cognitive impairment”[MeSH] AND (“amplitude of low frequency fluctuation”) |
| Web of Science | Same as Pubmed |
| Embase | Same as Pubmed |
